# Low serum uric acid levels are associated with the nonmotor symptoms and brain gray matter volume in Parkinson’s disease

**DOI:** 10.1007/s10072-021-05558-8

**Published:** 2021-08-18

**Authors:** Xiaoxue Shi, Jinhua Zheng, Jianjun Ma, Zhidong Wang, Wenhua Sun, Mingjian Li, Shen Huang, Shiyu Hu

**Affiliations:** 1 Department of Neurology, Zhengzhou University People’s Hospital, Zhengzhou, 450003 Henan Province China; 2 Department of Neurology, Henan Provincial People’s Hospital, Zhengzhou, 450003 Henan Province China; 3 Department of Neurology, Henan University People’s Hospital, Zhengzhou, 450003 Henan Province China

**Keywords:** Serum uric acid, Parkinson’s disease, Nonmotor symptoms, Brain gray matter volume

## Abstract

**Background:**

Uric acid (UA) plays a protective role in Parkinson’s disease (PD). To date, studies on the relationship between serum UA levels and nonmotor symptoms and brain gray matter volume in PD patients have been rare.

**Methods:**

Automated enzymatic analysis was used to determine serum UA levels in 68 healthy controls and 88 PD patients, including those at the early (*n* = 56) and middle-late (*n* = 32) stages of the disease. Evaluation of motor symptoms and nonmotor symptoms in PD patients was assessed by the associated scales. Image acquisition was performed using a Siemens MAGNETOM Prisma 3 T MRI scanner.

**Results:**

Serum UA levels in early stage PD patients were lower than those in healthy controls, and serum UA levels in the middle-late stage PD patients were lower than those in the early stage PD patients. Serum UA levels were significantly negatively correlated with the disease course, dysphagia, anxiety, depression, apathy, and cognitive dysfunction. ROC assessment confirmed that serum UA levels had good predictive accuracy for PD with dysphagia, anxiety, depression, apathy, and cognitive dysfunction. Furthermore, UA levels were significantly positively correlated with gray matter volume in whole brain.

**Conclusions:**

This study shows that serum UA levels were correlated with the nonmotor symptoms of dysphagia, anxiety, depression, apathy, and cognitive dysfunction and the whole-brain gray matter volume. That is the first report examining the relationships between serum UA and clinical manifestations and imaging features in PD patients.

## Introduction

Parkinson’s disease (PD), which has a prevalence rate of 5.42/1000 people, is a progressive neurodegenerative disorder that involves multiple neurotransmitter pathways [1, 2]. A diagnosis of PD depends on the presence of motor symptoms, including bradykinesia, rigidity, and tremor [3]. In addition to motor disturbances, PD patients also have other debilitating symptoms, which are classified as nonmotor symptoms [4].

Oxidative stress contributes to the loss of dopaminergic neurons in the substantia nigra of patients with PD and plays an important role in the pathogenesis of this disease [5–7]. As a natural antioxidant, uric acid (UA) can effectively scavenge reactive nitrogen and oxygen free radicals, so it plays a protective role in PD [8, 9]. Some studies have shown that serum UA levels are related to certain motor and nonmotor disturbances [4].

Among the existing methods, voxel-based morphometry (VBM) is a well-established structural magnetic resonance imaging (MRI) technique used to detect differences between groups in brain anatomy at the whole-brain level. VBM is a mature method for the evaluation of brain gray matter (GM) and white matter lesions that quantitatively calculates and analyzes the density and volume in the target area of an MRI image, reflecting the differences in the corresponding anatomical structures. Therefore, it provides an opportunity to discover structural changes that were previously unidentified, and the widespread use of VBM in the quantification of regional GM changes in PD has been encouraged [10].

To the best of our knowledge, there is currently no relevant literature exploring the relationship between serum UA levels in PD patients, nonmotor symptoms, and VBM. Therefore, this study aims to further explore the relationships between serum UA levels, nonmotor symptoms, and VBM and seeks evidence to support UA as a PD biomarker.

## Materials and methods

### Participants

A total of 89 PD patients from the inpatient ward were consecutively recruited from 2018 to 2020. Patients were diagnosed by two experienced neurologists, according to the UK PD Society Brain Bank Clinical Diagnostic Criteria for PD [11]. Patients with atypical and secondary PD or those who had been diagnosed with the following conditions that might interfere with serum UA levels were excluded as follows: (1) patients with cardiovascular and cerebrovascular diseases, such as myocardial infarction and cerebral infarction; (2) patients with acute or chronic infections or surgical procedures within the previous 3 months; and (3) patients with acute or chronic liver and kidney dysfunction or abnormal levels of serum creatinine (1.5 mg/dl) [12]. A total of 69 healthy volunteers participated in this study. All participants did not take any hormone treatment during the study.

### Clinical characteristics

General clinical data, such as sex and age, were recorded. The modified Hoehn and Yahr (H-Y) scale describes disease severity more broadly, with the early stage corresponding to H-Y stages 1 to 2, while the middle-late stage corresponds to H-Y stages 2.5 to 5 [13]. Motor symptoms were evaluated by Part III of the Unified Parkinson’s Disease Rating Scale (UPDRS) [14]. Nonmotor symptoms were evaluated by the Pittsburgh Sleep Quality Index (PSQI), Non-Motor Symptom Scale (NMSS), water swallowing test (WST), 14-item Hamilton Anxiety Rating Scale (HAMA-14), 17-item Hamilton Depression Rating Scale (HAMD-17), Modified Apathy Evaluation Scale (MAES), and Mini-Mental State Examination (MMSE). Hoehn and Yahr (H-Y) classification and the UPDRS were used to evaluate disease severity. All of the assessments were completed once during a patient’s off period.

### Blood sampling

Between 07:30 and 08:30 am and fasting serum UA concentrations were determined by an automated enzymatic assay.

### MRI acquisition

Image acquisition was performed using a Siemens MAGNETOM Prisma 3 T MRI scanner with a 64-channel head coil with the following parameters for the T1-weighted 3D-MPRAGE sequence: echo time (TE) = 3.43 ms, repetition time (TR) = 5,000 ms, inversion time (T1) = 755 ms, flip angle = 4°, slice thickness = 1.00 mm, slice number = 208, bandwidth = 240 Hz/pixel, a matrix of 256 × 256, field of view = 256 × 256 mm^2^, and voxel size = 1.0 × 1.0 × 1.0 mm^3^.

### Voxel-based morphometry analysis

The Gaussian default longitudinal preprocessing approach in the VBM8 toolbox was used with the following standardized steps: (1) registering the follow-up image to the baseline image for each subject; (2) calculating the mean image from the realigned images for each subject and using it as a reference image for subsequent spatial realignment; (3) correcting the realigned images for signal inhomogeneities with regard to the reference mean image; (4) performing tissue segmentation in the bias-corrected mean reference image and the bias-corrected realigned images; (5) estimating Diffeomorphic Anatomical Registration Through Exponentiated Lie Algebra (DARTEL) spatial normalization parameters with the tissue segments of the bias-corrected mean reference image; (6) modulating GM images to preserve relative regional volumes and correct for individual differences in brain size; (7) applying normalization parameters to the tissue segments of the bias-corrected realigned images; and (8) smoothing the resulting normalized tissue segments for each time point of each subject with an 8-mm full-width-half maximum (FWHM) Gaussian kernel [15].

### Statistical analysis

Quantitative data with a normal distribution in accordance with the Kolmogorov–Smirnov test are expressed as the means ± standard deviations, and Student’s *t*-tests were used for comparisons between the two groups. Multiple groups of data consistent with a normal distribution and homogeneity of variance were compared by one-way analysis of variance, and post hoc LSD *t*-tests were used to further compare differences in serum UA levels between the control group and the early stage and middle-late stage PD groups. Data that did not have a normal distribution are expressed as medians (quartile ranges), and the Mann–Whitney *U* test was used for comparisons. The identification of PD with dysphagia, anxiety, depression, apathy, and cognitive dysfunction based on serum UA was evaluated by receiver operating characteristic (ROC) curve analysis. Spearman’s correlation analysis was used to evaluate correlations between the serum UA levels and various indicators and the total GM volume in the brain. All tests were two-tailed, and a probability (*p*) value of less than 0.05 was considered statistically significant. The Statistical Package for the Social Sciences (SPSS) program version 26.0 was used for all statistical analyses.

To examine between-group differences in regional GM volume, the two-sample *t*-test was used to compare GM volume between the low-UA group and the high-UA group and was designed with age and sex as covariates. The statistical threshold was set at a cluster-level family wise error (FWE)-corrected *p* value < 0.05.

## Results

### Demographic data and serum UA levels in healthy controls and PD patients

**Fig. 1:**
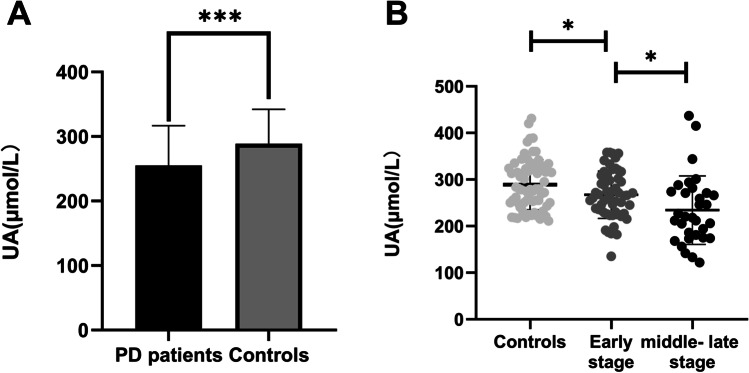
Differences in serum uric acid (UA) levels between Parkinson’s disease (PD) patients (*n* = 88) and controls (*n* = 68). **A** Serum UA levels in PD patients were significantly lower than those in controls. **B** PD patients were grouped based on the Hoehn and Yahr (H-Y) classification, and each of these groups was then compared to the controls. The early stage (*n* = 56) PD patients had lower serum UA levels than the controls, while serum UA levels were significantly decreased in the middle-late stage (*n* = 32) PD patients compared with the early stage PD patients. All data are presented as the means ± standard deviations. *p < 0.05, **p < 0.01, ***p < 0.001

No difference in age or sex was found between the PD and control groups (62.82 ± 6.36 vs. 62.71 ± 7.00, *t* =  − 0.105, *p* = 0.917; 52.9% vs. 44.9%, *t* = 0.972, *p* = 0.324), whereas serum UA levels in PD patients were significantly lower than those in healthy controls (Fig. 1A). Based on the H-Y classification, the PD patients were divided into early stage (*n* = 56) and middle-late stage PD patients (*n* = 32), and there were significant differences across the three groups (Fig. 1B). Serum UA levels in the early stage PD patients were lower than those in the healthy controls (*p* = 0.038), and serum UA levels in the middle-late stage PD patients were lower than those in the early stage PD patients (*p* = 0.010) (Fig. 1B).

### Correlations between serum UA and nonmotor symptoms in PD patients

UA levels were significantly negatively correlated with scores for the disease course, UPDRSIII, dysphagia, HAMA-14, HAMD-17, apathy, and NMSS and positively correlated with MMSE scores (Table 1). There was no correlation between UA levels and scores on the remaining scales (Table 1).

**Table 1:** Relationship between serum UA levels and demographic or clinical data in patients with Parkinson’s disease

	*Means* ± *standard deviations/medians (quartile ranges)*	*Pearson/Spearman rank*	*p values*
Age (y)	62.82 ± 6.36	− 0.206	0.055
Age of onset (y)	58.73 ± 9.32	− 0.116	0.281
Disease duration (y)	4 (2,7)	− 0.279	0.008*
UPDRSIII score	38 (21,50)	− 0.276	0.009*
WST score	3 (1,3)	− 0.503	0.000*
HAMA-14 score	15.45 ± 7.00	− 0.481	0.000*
HAMD-17 score	15 (10,20)	− 0.621	0.000*
MAES score	16 (8,23.75)	− 0.383	0.000*
MMSE score	25 (22,27)	0.506	0.000*
PSQI score	6.5 (3,11.75)	− 0.190	0.076
NMSS score	39 (29,67.75)	− 0.397	0.000*

Next, we used the significantly correlated nonmotor symptoms as grouping criteria for the PD patients to compare differences in UA concentrations between each pair of subgroups. This study included 88 patients with PD, 47 with MAES scores > 14 who had apathy (53.4%), 46 with WST scores ≥ 3 who had dysphagia (52.3%), 26 with HAMD-17 scores ≥ 20 who had depression (29.5%), 50 with HAMA-14 scores ≥ 14 who had anxiety (56.8%), and 58 with MMSE scores ≤ 26 who had cognitive dysfunction (65.9%).

**Fig. 2:**
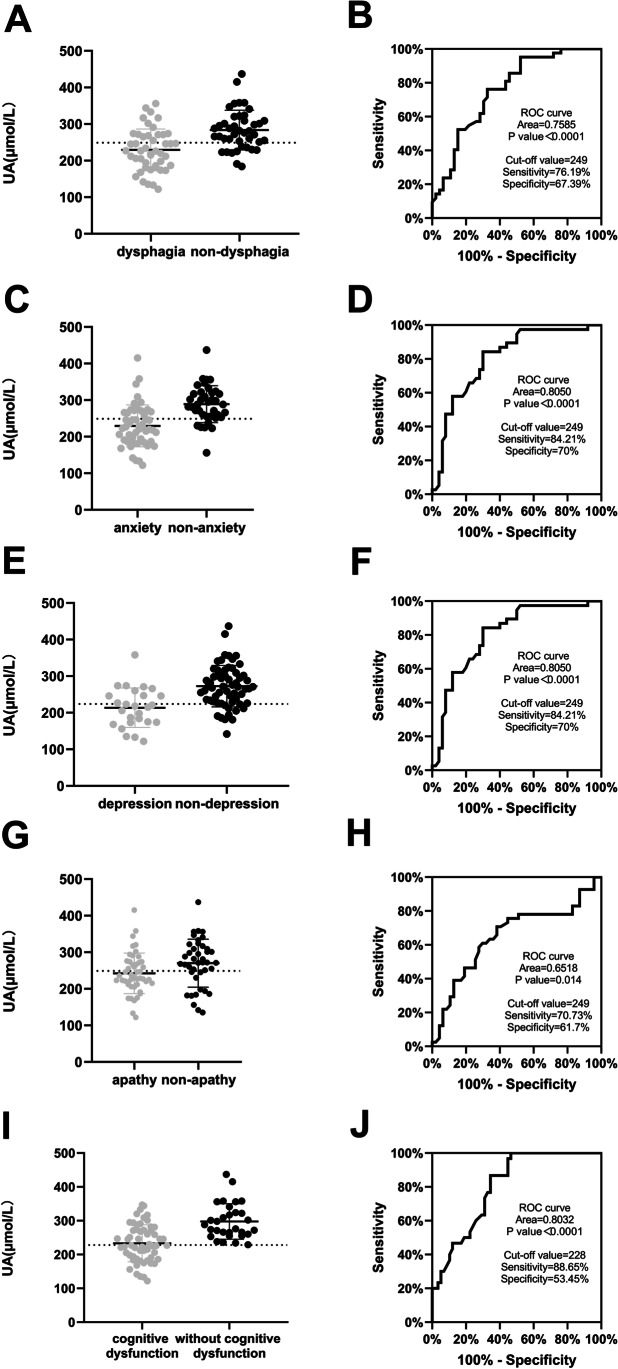
Relationships between serum uric acid (UA) concentrations and nonmotor symptoms in Parkinson’s disease (PD) patients (*n* = 88). **A** Serum UA levels in the dysphagia and non-dysphagia subgroups of PD patients. PD patients with dysphagia showed lower levels of serum UA. **B** Identification of PD with dysphagia based on serum UA shown by receiver operating curve (ROC) analysis. **C** Serum UA levels in the anxiety and non-anxiety subgroups of PD patients. PD patients with anxiety showed lower levels of serum UA. **D** Identification of PD with anxiety based on serum UA shown by ROC analysis. **E** Serum UA levels in the depression and non-depression subgroups of PD patients. PD patients with depression showed lower levels of serum UA. **F** Identification of PD with depression based on serum UA shown by ROC analysis. **G** Serum UA levels in the apathy and non-apathy subgroups of PD patients. PD patients with apathy showed lower levels of serum UA. **H** Identification of PD with apathy based on serum UA shown by ROC analysis. **I** Serum UA levels in the cognitive dysfunction and without cognitive dysfunction subgroups of PD patients. PD patients with cognitive dysfunction showed lower levels of serum UA. **J** Identification of PD with cognitive dysfunction based on serum UA shown by ROC analysis

Serum UA levels in the PD patients were lower in the dysphagia subgroup, anxiety subgroup, depression subgroup, apathy subgroup, and cognitive dysfunction subgroup than in the non-dysphagia subgroup, non-anxiety subgroup, non-depression subgroup, non-apathy subgroup, and without cognitive dysfunction subgroup (229.30 ± 57.12 vs. 283.64 ± 54.13, *t* = 4.570, *p* < 0.001; 229.52 ± 57.36 vs. 289.08 ± 50.40, *t* = 5.080, *p* < 0.001; 213.73 ± 54.02 vs. 272.65 ± 56.58, *t* = 4.515, *p* < 0.001; 242.30 ± 55.61 vs. 270.07 ± 65.70, *t* = 2.148, *p* = 0.035; 233.26 ± 54.60 vs. 297.73 ± 52.43, *t* = 5.322, *p* < 0.001, respectively). Based on a ROC curve analysis, identification of PD with dysphagia, anxiety, depression, apathy, and cognitive dysfunction based on serum UA had area under the curve (AUC) values of 0.7585, 0.8050, 0.7813, 0.6518, and 0.8032; sensitivities of 76.19%, 84.21%, 82.26%, 70.73%, and 88.65%; specificity of 67.39%, 70%, 61.54%, 61.7%, and 53.54%; and cutoff values of 249, 249, 224, 249, and 228, respectively (Fig. 2A–J).

*UPDRS*, Unified Parkinson’s Disease Rating Scale; *WST*, water swallowing test; *HAMA-14*, 14-item Hamilton Anxiety Scale; *HAMD-17*, 17-item Hamilton Depression Scale; *MAES*, Modified Apathy Evaluation Scale; *MMSE*, Mini-Mental State Examination; *PSQI*, Pittsburgh Sleep Quality Index; *NMSS*, Non-Motor Symptom Scale. *Statistically significant.

### Correlation between serum uric acid levels and gray matter volume in the whole brain

**Fig. 3:**
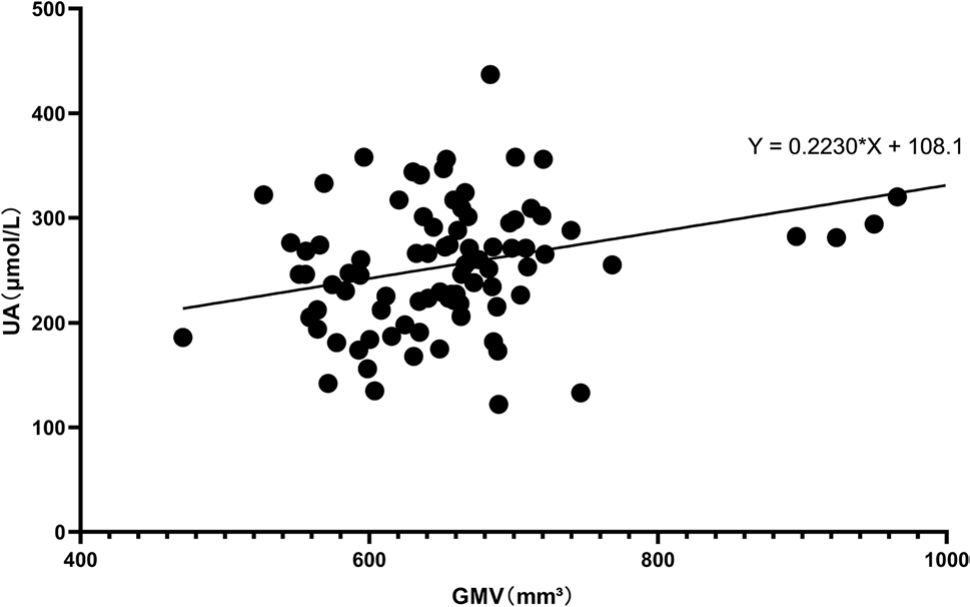
Relationship between serum uric acid (UA) concentrations and total brain gray matter (GM) volume. Serum UA levels were positively correlated with total brain GM volume (Pearson’s correlation coefficient *r* = 0.326, *p* = 0.002,* n* = 88)

The total brain GM volume in the PD patients with lower UA levels was significantly lower than that in the PD patients with higher UA levels. UA levels were significantly positively correlated with the GM volume in the whole brain (Fig. 3).

## Discussion

Studies have shown that UA levels are significantly related to the severity of dopaminergic impairment in the caudate, putamen, and striatum [16]. Therefore, we hypothesized that serum UA levels gradually decrease as PD progresses. Our study found a decrease in serum UA levels in PD patients. To investigate the relationship between serum UA levels and disease progression, we evaluated the association between UA levels and PD stages. After H-Y classification, the middle-late stage PD patients exhibited lower serum UA levels than the early stage PD patients. Subsequently, we explored factors that might have impacted serum UA levels. Correlation analyses showed that serum UA levels were negatively correlated with the course and severity of the disease. Similarly, previous studies have also proven that plasma or serum UA levels were lower in people with PD than in healthy controls [17, 18]. Furthermore, in postmortem substantia nigra tissue, UA levels were lower in patients with PD than in age-matched controls [19]. In a similar study published in 2016 conducted, the results showed that PD patients at stage three and over had significantly lower serum UA levels than PD patients at earlier stages [20] prospectively followed 804 PD patients and investigated the relationship between PD progression and serum UA levels, and the result was an inverse relationship between PD progression and serum UA levels, which is similar to what we observed in our study. It may be stated that there is an association of serum UA and disease progression. Adenosine, as a UA precursor, modulates neuronal death on its own, which reflects a neuroprotective effect [21]. Meanwhile, studies have proven that adenosine A_1_ and A_2A_ receptors induce either neuroprotective or neurotoxic effects on dopaminergic neurons [22, 23].

Nonmotor symptoms (NMS) manifest as cognitive, neuropsychiatric, autonomic, and sensory disturbances, which frequently worsen with disease progression [24], and the assessment and treatment of nonmotor symptoms may help improve the health-related quality of life of patients with PD [25]. In a large study of patients with PD, O’Sullivan et al. [26] suggested that NMS might be a significant feature in 21% of PD patients and that diagnostic delay and misdiagnosis were normal. Similar to previous findings, serum UA levels were negatively correlated with the severity of cognitive dysfunction [27–29]. Bowman et al. [30] proved that cerebrospinal fluid (CSF) UA and plasma UA levels were positively correlated and modified by blood–brain barrier (BBB) integrity and that CSF UA levels were associated with rates of cognitive decline. Study has confirmed an inverse correlation between UA levels and PD in the cortex and striatum, thereby supporting the theory that UA might have a neuroprotective effect on the cognitive system [31]. This may be because UA is both an antioxidant and an iron chelator, making it neuroprotective [32].

Neuropsychiatric symptoms, such as apathy, depression, and anxiety, are highly prevalent in PD patients and associated with decreased quality of life and adverse health outcomes [33]. Decreased serum UA levels have been found in PD patients with anxiety and depression [34]. A link between oxidative stress and emotional stress is not surprising, since it is well accepted that oxidative damage in the brain causes impairment of the nervous system [35]. Studies have proven that anxiety and depression are controlled by the nervous system and that the GABAergic and serotoninergic systems play important roles in the regulation of anxiety and depression [36]. Interestingly, we also found that the severity of apathy was positively correlated with serum UA levels. This correlation may be because both serum UA levels and apathy have been related to the loss of dopamine transporters (DAT) in the striatum [16, 37].

Dysphagia in PD patients has been associated with α-synuclein accumulation in the sensory nerve axons of the pharynx. Among them, the internal branch of the superior laryngeal nerve is the most involved. At the same time, it has also been found that there is α-synuclein in the efferent pathway that innervates the pharyngeal muscles [38, 39]. Therefore, the significantly lower UA levels in PD patients with dysphagia may further damage the nerves that innervate swallowing function, leading to the occurrence of dysphagia symptoms. Therefore, we suspect that nonmotor symptoms, such as cognitive dysfunction, anxiety, depression, apathy, and dysphagia, which often occur in the middle and late stages of PD, may be another possible potential cause for decreased serum UA levels. However, the correlations among the mechanisms of PD, serum UA levels, and nonmotor symptoms need to be further explored.

Finally, we explored the relationship between brain volume changes and serum UA levels in PD patients. We found that serum UA levels were positively correlated with total brain GM volume. We did not find a relationship between specific GM areas of the brain and serum UA levels. We considered the following reason for this conclusion in PD patients. Studies have proven that serum UA levels are negatively correlated with the severity of PD [40]. At the same time, studies have confirmed that in early PD patients, global GM loss, amygdalar atrophy, and cortical thinning in frontotemporal regions are specifically associated with the PD degenerative process [41]. UA plays a neuroprotective role in dopaminergic neurons by regulating neuroinflammation and oxidative stress [42]. Oh et al. proved that UA levels were positively correlated with dopamine transporter uptake in the putamen in female early PD patients, and this finding suggested that UA had a neuroprotective effect, as evidenced by the relatively preserved striatal dopamine activity in women [43]. Therefore, we speculate that UA, as a protective substance in PD, has a broad protective effect on brain neurons. As UA decreases, its protective effect decreases. Therefore, we concluded that, in PD, lower UA levels are associated with a decrease in brain volume compared to volumes in those with higher UA levels.

This study has the following limitations. First, this study proposed that lower UA levels in PD patients caused a decrease in the whole-brain GM volume and testing this will require repeated testing and an expanded sample size for further verification. Second, the Non-Motor Symptom Scale was evaluated, which might be affected by subjective factors; therefore, more objective evaluation methods need to be used, such as video fluoroscopy studies of swallowing (VFSS), to verify these results. Finally, although our data showed that serum UA levels gradually decreased as the course of the disease progressed, this was a cross-sectional study. Therefore, longitudinal studies are needed to clarify the relationships between the development of PD and serum UA levels in the presence of various confounding factors.

## Conclusions

This study shows that serum UA levels can be used to assess the possibility of PD with nonmotor symptoms, such as cognitive dysfunction, anxiety, depression, apathy, and dysphagia, and are positively correlated with the whole-brain GM volume. These findings indicate that monitoring serum UA levels may be a potential biomarker or treatment strategy for PD. Finally, to clarify the clinical significance of serum UA concentration in PD patients, larger clinical and preclinical studies are needed to further explore the potential mechanism underlying changes in serum UA levels in PD.
